# Topics of Public Interest

**Published:** 1909-03

**Authors:** 


### TOPICS OF PUBLIC INTEREST.

### How Infection Affects the Body

BY STEPHEN SMITH, M.D., LL. D.

[*New York Tribune Sunday Magazine.*]

HOW infection affects the body was the supreme mystery that the scientists of the past strove in vain to penetrate. By no devices of their laboratories could they detect the agents that caused an epidemic. There was only one satisfactory explanation of the origin and spread of the devastating plagues which seemed to fall from the heavens on the people, and that was that epidemics were "a visitation of God" on account of the sins of the people. Of course the only preventive and curative measure available and effectual was "repentance, prayer, and humiliation."

It is a cause of devout thankfulness that while these things were hid from the "wise and prudent" of former times, they have in these latter days been revealed unto "babes." No event in human history would have more greatly taxed the credulity of the most learned and experienced physician of half a century ago than the prophecy that in the early years of the twentieth century school children would be taught by simple and easily understood object lessons how to prevent and how to cure consumption, the Asiatic cholera, yellow fever, and other epidemics that have devastated cities, destroyed armies, and swept from the earth whole tribes of primitive people.

But that prophecy has been literally fulfilled. During the last summer there has been a traveling object lesson that visited the differnt sections of the State of New York and taught the people, especially the children, all of the essential facts as to the nature of the infection of tuberculosis, its effects on the body, and the methods of prevention and cure.

As infective diseases cause the vast majority of cases of severe and crippling affections and of deaths in every community, the value of a knowledge of the nature of infection and how it affects the body, by the people of all ranks, ages, and conditions, cannot be estimated in its influence on the future of the human race. Already we learn that within the period referred to the sickness and death rates of communities where the people have been most thoroughly instructed as to the nature of infective diseases and how they affect the body, have greatly diminished, and the average of human life has been markedly lengthened. Indeed, it now seems possible to restore the patriarchal age when a man may live to be "an hundred and twenty years old . . . his eye . . . not dim, nor his natural force abated."

To understand how infection affects the body involves an inquiry as to the nature of infection, its mode of entrance into the body, and its operations on its organs and tissues. The terms "infection" and "contagion" are often used as synonymous; but a strict definition according to the medical significance of each limits the former to "the transmission of disease by actual contact of the diseased part with a healthy absorbent or abraded surface," and the latter to "transmission through the atmosphere by floating germs." But in the final analysis the cause of disease in both infection and contagion is so similar in its action that the medical profession has adopted the term "communicable disease" in all cases where the disease is communicated from one person to another by means of a germ, whatever may be its method of attack on the body.

### WHAT THE GERM IS.

What is this communicable germ or agent? A bacterium—a little stick, staff—so called from the rodlike shape it assumes in the process of growth. The individual bacterium (plural, bacteria) is an organism representing a low form of vegetable life; resembles mold; in size the smallest living thing that can be seen with the microscope; in masses forms the films floating on foul fluids or covering decomposing animal or vegetable matter. It consists of a single cell, and its mode of increase when placed under proper conditions for growth is by division of the cell body; the two cells formed out of the first being divided into four before complete separation has taken place; the four dividing into eight, the eight into sixteen, the sixteen into thirty-two, and so on indefinitely. Now, as it requires only thirty minutes for one cell to divide, it has been estimated that a single bacterium will in twenty-four hours increase to the number of over sixteen million five hundred thousand, and in forty-eight hours to two hundred and eighty-one million five hundred thousand. At this rate of increase, in three days there would be a mass of bacteria weighing about sixteen million pounds. As the multiplication of bacteria depends upon conditions that soon interfere with or interrupt their growth, as the want of food, their own secretions, and certain natural forces operating against them, these stupendous figures are useful only as an illustration of the enormous fertility of these organisms, and their destructive energy when they attack a susceptible living body.

What is the function of bacteria in the economy of nature? It would be surprising if such a menace to human life as some species of bacteria have proved themselves to be had no other place among the forces of nature than to prevent the too rapid increase of the human race on this earth, as our forefathers believed. It is gratifying, and quite satisfying to a revengeful spirit, to learn from the modern laboratory that the special and only function of the bacterium is to perform the duties of a universal scavenger. It is always seeking decomposing animal and vegetable matter. It lives on filth, riots in it, and dies when deprived of it. It enters the human body only in search of filth, and if it finds none it does the person no harm, and dies either from the want of food or by starvation, or escapes from the body, or secretes itself where it may safely await the creation of decomposing matter, when it will begin its lifework.

Thus, there may be and doubtless is at all times a great variety of bacteria of a virulent type quiescent in our bodies only for the time that they find no decaying matter adapted to their special tastes or wants.

It is a most interesting fact, therefore, that this most deadly foe of man becomes dangerous only when the latter is harboring in his body waste or decomposing matters that are slowly poisoning him. It is in the process of digesting this material that the bacterium excretes poisons—toxins—of the most virulent nature, which are absorbed into the blood of the human victim, creating the condition popularly known as blood poisoning.

Bacteria perform a most important function in the economy of nature; *viz.*, the conversion of decaying and dead matter into food for plants. Biologists assert that without bacteria plant life on the earth would be scanty or entirely wanting; they are the natural intermediaries between plants and animals in point of food production. They are therefore called scavengers, because they live on decomposing matter; but in the very act of digesting such waste they convert it into produces essential to plant life (carbon dioxide and ammonia) and by their excretions restore to vegetation its chief supply of food.

It appears on the same authorities that bacteria not only assist materially in maintaining vegetable and animal life on this planet, but "in the arts and industries they are as essential to modern economic life as are the ingenious mechanical inventions of men. Many secret processes now in use in the arts and manufactures are but devices to harness these natural forces. Thus in the manufacture of linen, hemp, and sponges, in the butter, cheese, and vinegar industries, in tobacco curing, etc., bacteria play an important role.

### BACTERIA FOR EVERY CONDITION.

It naturally occurs that to meet the various conditions under which decomposing matter exists in nature there is a great variety of species of bacteria, each species being adapted to a special field of operations. These species are distinguished from one another by the shapes they assume during their growth, some being rod shaped (the bacillus), others spherical (the coccus), and others spiral (the spirillum). Under one of these divisions the various species are classified.

In these latter days of popular knowledge of scientific progress, but without precise information of details, bacteria are associated in the public mind with disease, especially of the epidemic form. While this prejudice is useful in stimulating the people to adopt and enforce preventive measures against conditions that tend to promote bacterial life in their homes and in their own persons, yet it should be understood that comparatively few of the great number and variety of bacteria are pathogenic, or disease producing, in man.

So throughout the animal kingdom we find that few are susceptible to a common disease; or, in other words, that the same species of bacteria attack with equal force several varieties of animals.

The explanation of this peculiarity is found in the variations of the quality or intimate nature of the tissues and organs of different species of animals. The same may be said of our own bodies,—the several organs vary greatly in their susceptibility to the attacks of the different kinds of bacteria; hence, the latter are classified as specific and nonspecific, according as they cause specific or nonspecific disease.

The distribution of bacteria is limited only by the existence of plants and animals; that is, the existence of decomposing vegetable and animal matter. Though they are more abundant in the earth where such matter is found most abundantly, yet they abound in the air, the water, on plants, animals, and insects, on our own bodies, and in every cavity leading to the exterior. As bacteria are always searching for food, the number present is a sure indication of the degree of cleanliness of the thing, individual, or locality where they are found.

The movements of bacteria from one point to another are through the medium of some other mode of conveyance than their own bodies afford. Thus they are borne by the water, by vegatation, by animals of every kind especially insects, by the air on particles of dust. The typhoid bacillus borne in water and milk has caused innumerable epidemics of that dreaded disease.

### THE DEADLY TUBERCLE BACILLUS.

The tubercle bacillus is borne on the air through the medium of particles of dust, and in cities where the victims of tuberculosis scatter these germs profusely in the streets, public conveyances, churches, and places of resort, in the act of coughing, sneezing, and spitting, the dust borne on the winds is a constant and most fertile source of infection of tuberculosis. In a city like New York thousands are annually infected by the dust borne tubercle bacilli, not only by inhaling them in the street, but even more certainly in the quiet of their homes, where the germ bearing dust accumulates in clothing, bedding, carpets, rugs, and upholstered furniture, and is daily forced into the air of the living rooms by broom and duster.

Foul as is the air of the unventilated tenements of the poor, it has been demonstrated that the dust which saturates the furniture, carpets, rugs, and hangings of the residences of the wealthy contains sixty per cent. of ordinary street filth.

An authority says: "The most widely distributed pathogenic microörganism (disease causing bacterium) in the air is the tubercle bacillus, the cause of consumption and a large variety of other ailments, such as hipjoint disease, caries of the spine, etc. Over one hundred thousand persons die annually from consumption alone in the United States, and it is estimated that there are over two million people afflicted with the disease in one form or another. All of these sufferers are expectorating billions of tubercle bacilli daily."

### HOW THEY AFFECT THE BODY.

Considering the second inquiry as to how infection affects the body, we must constantly bear in mind that a bacterium, though a scavenger, is a conservator of nature. Its real function in the orderly processes of animal and vegetable life is to utilise waste for the preservation and promotion of animal and vegetable life on this planet where the conditions are so unfavorable to both.

Therefore, wherever we find bacteria in the active process of growth, that is, multiplication, we may be assured that they have found matter that should be rescued from waste and converted into useful food for plants. It follows that when we find a bacterium actively growing in any part of our bodies, it has found some form of decaying matter that is not only no longer useful to our bodies, but is in fact harmful and should be removed.

It is also important to understand that waste matter is found under a great variety of conditions, and that for its proper conversion into useful food for plants there must be a correspondingly large number of species of bacteria each having its special field of operation. It is due to this variety of bacteria that there are so many infective diseases; for each species of bacteria creates its own individual form of disease.

This statement requires the following explanation; *viz.*, a bacterium in a quiescent state is harmless; everyone has within his body innumerable bacteria, as the tubercle and typhoid bacilli; but they are inert, and hence innocuous. It is only when they find their proper food, decaying matter, that they begin to multiply, and in that act they secrete a poison, toxin, which is absorbed and, entering the circulation, causes in the individual a special class of symptoms peculiar to that toxin, or poison.

These symptoms constitute a disease, the technical name of which is usually fanciful, depending on some feature of the symptoms, but explaining nothing as to its essential nature.

For example, the typhoid bacillus finds its food in certain minute glands of the small bowels. If these glands are in a perfectly healthy state when the bacillus enters the digestive tract, the germ will pass over them and disappear from the body perfectly harmless. But if the bacillus finds its appropriate food—dead or decomposing matter—in the glands, it at once takes up its abode in them and "begins housekeeping;" that is, it begins to multiply according to the method of fission of its cell and rate of multiplication, already described. During this process the multiplying cells excrete a toxin, which, being absorbed, creates a fever, the result of a true blood poisoning. This fever is called typhoid, because its prominent symptom, stupor, resembles that of typhus fever. The name, therefore, signifies nothing as to the nature of the disease.

### KEEP IT OUT OF THE CIRCULATION.

The poisoning of the body by the excreted toxin of the multiplying cells, which is simply plant food, occurs because it is removed only in part by the digestive organs, the circulation that conveys it to the other eliminating organs being efficient for that purpose. Could all of this toxin be removed as fast as it is excreted, and not enter the circulation, there would be no fever.

The termination of this process must be either the death of the colony from exhaustion of the food supply in the glands, or the exhaustion of the patient by the excess of toxins that accumulate in the body. As the activity of the bacillus depends upon the food supplied, the severity and length of the fever varies in different individuals. Some are immune because the glands that furnish the food of the typhoid bacillus are in a state of high health; others have a brief and mild attack, because the food supply is scant owing to a slight impairment of the integrity of the glands; but with a considerable number in every epidemic the food is ample to sustain the creation of an immense colony of bacilli which destroys the victim by an overdose of poison.

The final disposition of the typhoid bacilli after a course of fever, was believed to be by their elimination from the body through the various organs devoted to the discharge of waste products; but recent investigations have proved that the typhoid bacillus may remain in the body for long periods without apparently affecting the health of the person, but when communicated to another it will cause an attack of fever of the most virulent type. In one instance an outbreak of typhoid fever was traced to a woman who had the fever upward of fifty years ago. It was found that the excretions of her body contained immense quantities of living typhoid bacilli. She was a cook by trade, and it was found on tracing her history that wherever she had worked there had been epidemics of typhoid, beginning at the house where she was acting as a cook.

A still more remarkable feature of the life history of the typhoid bacillus has recently been made public. A typhoid epidemic was traced to a nurse who had attended cases of typhoid fever, but had never suffered from an attack of that disease, and yet was discharging large quantities of the bacilli. These cases can be explained only on the theory that these microörganisms find some place, possibly, as has been suggested, in the gallbladder, where they find food sufficient to keep them in an active state of multiplication, but where the conditions prevent the absorption of the toxins they excrete.

How far these curious incidents in the life of the typhoid bacilli are common to other bacilli is not known; but if it is true of other infectious diseases, the fact will explain the origin of those obscure and mysterious cases that occur without any known exposure to the infection.

AIMS TO DESTROY THE BODY.

In concluding this inquiry as to the nature of infection and its effects on the body, the following statement of a biologist as to the bacterium seems justified: "When it enters a living body, it aims directly at the destruction of the latter. It multiplies rapidly, tends to scatter its broods throughout the tissues, and all the while gives off the most powerful poisons. This agent is wickedly implacable, neither giving or asking quarter. The battle that it wages with the body can terminate only by the destruction of one of the combatants."

Viewed in the light of the past history of infectious diseases, this is not an overdrawn picture. If we estimate the deaths from smallpox in ancient times, from cholera in modern times, and from tuberculosis (consumption) throughout all time, the destruction of human life by bacteria cannot be overstated. The bacterium has been a wickedly implacable foe to the human race in the past. Invisible, intangible, everywhere present, it has proved omnipotent in its destructive attacks upon communities.

But our century opens with a far brighter outlook for the race. Elementary forces which, through ignorance of their true functions in the economy and conservatism of nature, were permitted in the past to expend their energy in the destruction of life, have been revealed by science to be man's most helpful agents in the promotion of comfort, health, and longevity. Electricity was for ages only a thunderbolt, an object of terror and an agent of destruction, visiting the human residence only to kill its owner and burn the structure.

Today the same natural force is man's most obedient and humble servant, quietly visiting his home to furnish him heat and light, annihilating time in the transactions of business, and transporting him from place to place as on the lightning's wings.

So the bacterium, once the terror of mankind as the invisible and apparently unknowable cause of devastating pestilences, proves to be the useful purveyor of the by-products of its digestion of waste matter which is thereby converted into food for plants. It visits man in the pursuit of its humble calling to obtain his contribution to the sum total of plant food. It searches every tissue, every organ, every recess, however obscure, but so stealthily that its coming and going and its immediate presence are not known if absolute cleanliness of the body exists. It is only when dying tissues or organs, or accumulations of dead matter, are found that its presence becomes known. Even then it would prove harmless and its presence would be unrecognised if its excretions of plant food (toxins), were not necessarily absorbed and did not enter the circulation, thus poisoning the body it is relieving of dead matter.

MAN'S DEFENSES.

Briefly, what are man's defenses against bacteria?

Chiefly two, *viz.*, first, killing it by depriving it of food; and, second, killing it directly by what are known as germicides.

The first method is effected by cleanliness of the person. It may be affirmed that cleanliness, without and within, absolutely protects every man, woman, and child from the most common disease-producing bacteria. It is not sufficient to keep the skin clean by daily baths, while the mouth, nose, throat, and other internal surfaces and organs are covered or filled with effete matter. We must be every whit clean if we would escape the results of the scavenging processes of bacteria of some variety or species.

That condition can be secured and maintained in an organism that itself is constantly decaying in all of its tissues and organs only by strict compliance with the natural laws governing the operations of the body as an independent organism in which all of its forces tend to promote its health and conservation. Every tissue and every organ has its special means of renewal of its tissue by the removal of dead particles through the outlets and the reception of fresh material through the inlets of the body. Waste and supply are exactly balanced, as in the most precise and delicate machine. If the outlets become clogged so that all the waste cannot escape at the proper time, dead matter, the food of bacteria, begins to accumulate, and disease must be the result.

In the same manner, if the food is in excess of the demands, or of a quality not suited to the needs of the tissue or organ, waste begins to accumulate, bacteria swarm in the decomposing mass, and emit their toxins, which, absorbed into the circulation, cause a variety of physical disturbances according to the species of bacteria present, and the particular tissues the toxins affect, as the nervous system, stomach, heart, kidneys, etc.

That even the most feeble minded may be able to regulate their habits so as to secure an adequate supply of food both in quality and quantity, and the prompt removal of waste matter, so as to secure that degree of cleanliness of internal organs essential to escape from bacterial attacks, the mechanism of the body is endowed with instincts that make it automatic in its action. Such are appetite and taste for food and drinks; the desire for exercise, rest, and sleep; the impulse of the organs in an active state; etc. It is only when these natural monitors are interfered with that the mechanism begins to fail in its elimination of waste, and bacteria find the conditions favorable for their functional activity.

### DESTROY THE BACTERIA.

The second defensive measure is the destruction of the bacteria by means of agents that will destroy the microörganism before or after its entrance into the body, but without injuring the healthy tissues. There is a great variety of these agents of more or less power, and they are used in the form of gases, liquids, and powders, according to conditions existing in individual cases. In general, it may be advised that, as bacteria are everywhere, germicides ought to be used far more extensively than they are for the purposes of securing not only the direct destruction of bacteria, but of removing or neutralising dead matter, the food of bacteria. So minute are bacteria, and so adherent are they to material things, that mere bathing with water does not remove them, medicate it as we may with fancy soaps. There should be used in addition a more penetrating and destructive agent, which would not only destroy all forms of bacteria, but at the same time secure absolute cleanliness.

